# Identification of a complex genomic rearrangement in *TMPRSS3* by massively parallel sequencing in Chinese cases with prelingual hearing loss

**DOI:** 10.1002/mgg3.685

**Published:** 2019-04-23

**Authors:** Xinlei Li, Bo Tan, Xiaoqian Wang, Xiaofei Xu, Cuicui Wang, Mingjun Zhong, Qiuling Zhao, Zhongwei Bao, Weihua Peng, Lei Zhang, Jing Cheng, Yu Lu, Peina Wu, Huijun Yuan

**Affiliations:** ^1^ Medical Genetics Center Southwest Hospital, Army Medical University Chongqing China; ^2^ Department of Otorhinolaryngology Head and Neck Surgery Guangdong Provincial People's Hospital, Guangdong Academy of Medical Sciences Guangzhou China

**Keywords:** copy number variation, hearing loss, massively parallel sequencing, rearrangement, *TMPRSS3*

## Abstract

**Background:**

Genetic variants in *TMPRSS3* have been causally linked to autosomal recessive nonsyndromic hearing loss (HL) at the DFNB8 and DFNB10 loci. These variants include both single nucleotide and copy number variations (CNVs). In this study, we aim to identify the genetic cause in three Chinese subjects with prelingual profound sensorineural HL.

**Methods:**

We applied targeted genomic enrichment and massively parallel sequencing to screen 110 genes associated with nonsyndromic HL in the three affected subjects. CNVplex^®^ analysis and polymerase chain reaction (PCR) were performed for CNV detection.

**Results:**

We identified biallelic variations in *TMPRSS3* including a novel complex genomic rearrangement and a novel missense mutation, c.551T>C. We have mapped the breakpoints of the genomic rearrangement and showed that it consisted of two deletions and an inversion encompassing exon 3 to exon 9 of *TMPRSS3*.

**Conclusion:**

Our study expanded the mutational spectrum of *TMPRSS3* to include complex genomic rearrangements. It showcased the importance of an integrative approach to investigate CNVs and their contribution to HL.

## 1 INTRODUCTION

Hearing loss (HL) is the most common sensory deficit, affecting one to two per 1,000 newborns (Morton & Nance, 2006; Sloan‐Heggen et al., 2016). Approximately 60% of HL is caused by genetic factors (Morton & Nance, 2006). Nonsyndromic hearing loss (NSHL), in which hearing impairment is the only obvious clinical abnormality, accounts for 70% of the genetic cases (Gao et al., 2017). Autosomal recessive nonsyndromic hearing loss (ARNSHL) is the most common type in NSHL, usually manifested as severe to profound, prelingual and nonprogressive HL (Petersen & Willems, 2006).

The *TMPRSS3* gene (OMIM 605511) encodes a transmembrane (TM) protease protein containing TM, low‐density‐lipoprotein receptor A (LDLRA), scavenger‐receptor cysteine‐rich (SRCR), and serine protease domains (Scott et al., 2001). Mutations in *TMPRSS3* are associated with both prelingual ARNSHL (DFNB10) and postlingual ARNSHL (DFNB8) (Bonne‐Tamir et al., 1996; Veske et al., 1996). Mutations in *TMPRSS3* are suggested to be classified as mild and severe ones based on their corresponding phenotypic effects (Weegerink et al., 2011).

Copy number variation (CNV) is widespread in human genome and represents a significant source of genetic variation (Zhang, Gu, Hurles, & Lupski, 2009). Copy number variation is a well‐recognized cause of genetic diseases through various molecular mechanisms, including gene dosage, gene disruption, gene fusion, position effects, etc. (Zhang et al., 2009). Previous comprehensive genetic tests performed on patients with NSHL indicated that copy number variant was an important cause of NSHL (Shearer et al., 2014).

In this study, we conducted targeted genomic enrichment, massively parallel sequencing (MPS) and quantitative analysis in three cases with prelingual profound HL and identified biallelic variations in *TMPRSS3*, including a complex genomic rearrangement and a missense mutation.

## 2 MATERIALS AND METHODS

### 2.1 Subjects

This study was approved by the Ethics Committee of First Affiliated Hospital of Third Military Medical University (Army Medical University). Three Chinese subjects in two families with prelingual profound sensorineural HL were recruited. The severity of deafness was defined as profound (>90 dB HL) based on the thresholds of pure‐tone audiometry. A total of 300 subjects with normal hearing were recruited as a control group. Peripheral blood samples and clinical information were collected from subjects and their family members if available. Written informed consents were obtained from the participants or their parents.

### 2.2 MPS and bioinformatic analysis

Total human genomic DNA was isolated by the AxyPrep‐96 Blood Genomic DNA Kit (Axygen Biosciences, Union City, CA). Prior to MPS, screening on common variants in *GJB2*, *SLC26A4,* or *MT‐RNR1* was conducted and no causative variants were detected in the three participants. Massively parallel sequencing covering 110 NSHL associated genes was then completed in the subjects using Agilent SureSelect Target Enrichment Kit (Agilent Technologies, Santa Clara, CA) and Illumina HiSeq 2000 System (Illumina, San Diego, CA) as described (Wang et al., 2017).

Sequence data were analyzed using a custom variant analysis workflow. Raw sequence reads were mapped to the human reference genome (GRCh37/hg19) using Burrows‐Wheeler Aligner (version 0.7.15), followed by variants calling using Genomic Analysis Tool Kit best practices. Variants were annotated using Variant Effect Predictor and filtered for minor allele frequency (MAF) in gnomAD and variant consequence. In silico predictions for conservation (PhyloP and GERP++) and functional effects [SIFT (Sorting Intolerant From Tolerant), Polyphen‐2, LRT, MutationTaster, and CADD (Combined Annotation Dependent Depletion)] were used to assess variant conservation and predicted deleteriousness. Molecular modeling of wild‐type and mutant structures of *TMPRSS3* were based on the tertiary structure of the TM protease acquired from SWISS‐MODEL (https://swissmodel.expasy.org/) and presented using Pymol‐v1.3. Pathogenicity of the variants was analyzed according to the recommendations for the interpretation of sequence variants of American College of Medical Genetics and Genomics (ACMG) (Richards et al., 2015).

Massively parallel sequencing reads were visualized by Integrated Genomics Viewer 2.4.10 using sample bam files. We used the UCSC (University of California, Santa Cruz) BLAT (Blast‐Like Alignment Tool) Search Genome tool (http://genome.ucsc.edu/cgi-bin/hgBlat?command=start) for genomic sequence alignment.

### 2.3 CNVplex^®^ analysis

CNVplex^®^ technique (Zhang et al., 2015), a high‐throughput multiplex CNV analysis method developed by Genesky Biotechnologies (Shanghai, China), was used to analyze the copy number of *TMPRSS3* (NM_024022.2) in GD‐395. Fifty probes were selected including 26 target‐specific probes and 24 reference probes located at different subchromosomal loci, which had not been reported to have any copy number polymorphisms. Two probes were designed for each exon and probes targeting 10 and 2 kb upstream of *TMPRSS3* were also included (Table S1).

### 2.4 Real‐time Polymerase chain reaction (PCR)

PCR primers (Table S2) were designed for seven exons and two introns of *TMPRSS3*. *COBL* and *RPP30* were selected as endogenous controls in this study. Real‐time PCR of GD‐395 was carried out on the 7500 Fast Dx Real‐Time PCR Instrument (Applied Biosystems, Foster City, CA). PCR amplification (10 μl) was carried out using QuantiNova™ SYBR^®^ Green PCR Kit (Qiagen, Hilden, Germany) at 95°C for 2 min, followed by 40 cycles at 95°C for 5 s and 60°C for 30 s. The pre‐experiments for each primer were conducted for a standard curve from a set of diluted standard DNA to ensure the efficiency and specificity of PCR amplification. In each assay, samples and two normal controls were included in triplicate for each primer. For data analysis, the relative copy number was determined by the comparative C_T_ method.

### 2.5 Long‐range PCR, gap‐PCR, and Sanger sequencing

Long‐range PCR of GD‐395 was performed on 2720 Thermal Cycler (Applied Biosystems) using the TaKaRa LA Taq^®^ Hot Start Version (Takara Bio, Otsu, Japan). Approximately 50 ng of high quality template DNA was added to a 15 ul standard reaction. The forward primer (TMPRSS3_In2_F) and the reverse primer (TMPRSS3_Ex12_R) were added to a final concentration of 0.67 μmol/L. Thermocycling conditions were as follows: 1 cycle of 95°C for 5 min, 30 cycles of 98°C for 20 s and 68°C for 12 min, and 1 cycle of 68°C for 7 min. Gap‐PCR was designed to detect the certain CNV in single PCR amplification for GD‐395 and his family members. One reverse primer (TMPRSS3_Ex12_R) and two forward primers (TMPRSS3_In2_R and TMPRSS3_Ex11_F) were added to a single gap‐PCR amplification. Standard protocols of Sanger sequencing were followed on the ABI 3500xL Dx Genetic Analyzer (Applied Biosystems) to confirm detected variants in cases and extended families.

## 3 RESULTS

### 3.1 Case presentation

**Figure 1:**
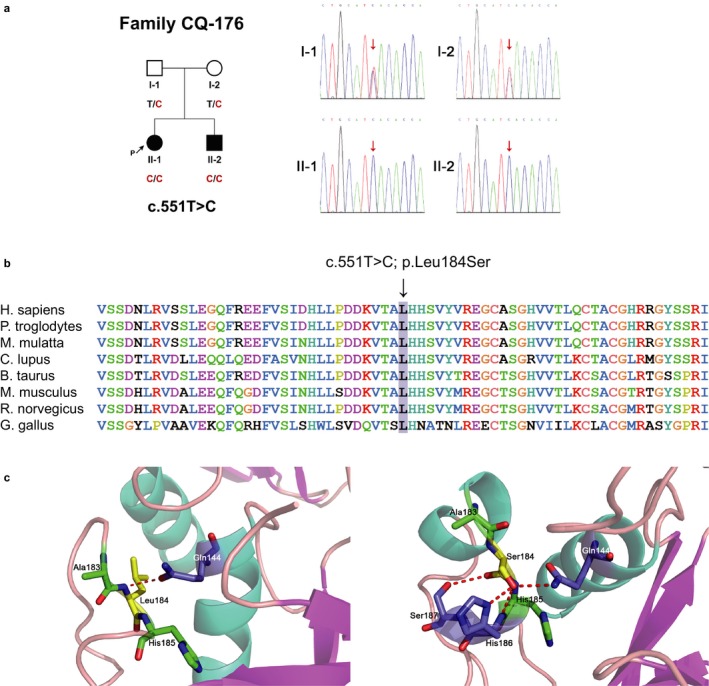
(a) Pedigree and Sanger sequencing of Family CQ‐176. In the pedigree, black and white symbols represent people with hearing loss and normal hearing, respectively. The genotypes are labeled below. (b) Conservation analysis: the residue Leu184 is highly conserved among eight different species. (c) 3D structure prediction: the structure of the wild‐type protein and the p.Leu184Ser protein. Red dotted lines indicate the hydrogen bonds between the 184th residue and other residues

**Figure 2:**
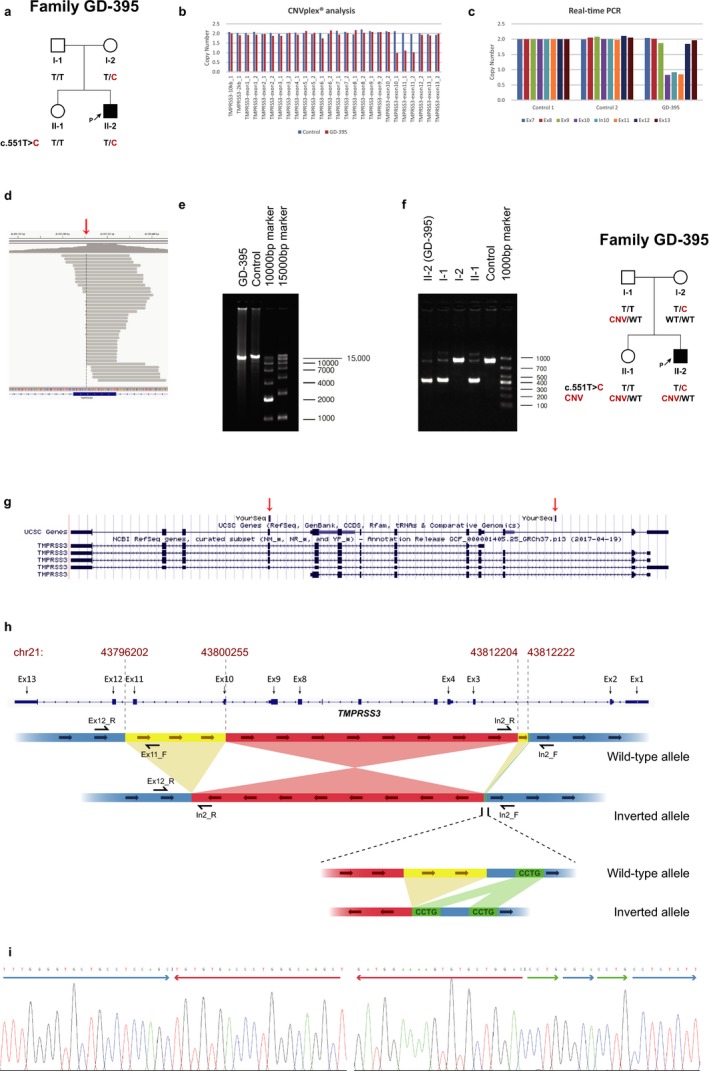
Identification of a novel complex genomic rearrangement in *TMPRSS3*. (a) Pedigree of family GD‐395. (b) The copy number of each exon calculated from the fluorescence peak ratios in CNVplex^®^ analysis. (c) The copy number of the exons of interest from real‐time PCR. (d) The drop of read‐depth and the split reads in exon 10 of *TMPRSS3*. (e) Long‐range PCR conducted by TMPRSS3_In2_F and TMPRSS3_Ex12_R. The inverted allele generated a product of 12K, while the normal allele came to a 16K. Only the inverted allele could be amplified in GD‐395. (f) Gap‐PCR and segregation analysis of family GD‐395. (g) The genomic alignment of one of the split reads conducted by UCSC BLAT. (h) Scheme of the normal and inverted alleles. (i) Sanger sequencing of the inverted allele by TMPRSS3_In2_F and TMPRSS3_Ex12_R covering the two breakpoints

Two families ascertained for this study segregated ARNSHL (Figures 1a and 2a, Table 1). Individuals CQ‐176‐II‐1 and CQ‐176‐II‐2 were siblings with congenital profound deafness. GD‐395 was a 3‐year‐old male, which was first sent for audiometric testing upon parents reporting failure to respond to loud noises. Auditory brainstem response testing revealed bilateral profound sensorineural HL across all frequencies. Computed tomography analysis of GD‐395 ruled out the presence of inner ear malformations. Comprehensive family medical histories and clinical examinations of these three individuals showed no other clinical abnormalities, including vestibular defects, diabetes, cardiovascular diseases, visual problems, and neurological disorders.

**Table 1:** Summary of phenotypic and genotypic information for the three subjects

Sample	Sex	Age at test (years)	Age of onset	Severity	Variant 1	Variant 2
GD‐395	Male	3	Prelingual	Profound	p.Leu184Ser^a^	Genomic rearrangement
CQ‐176‐II‐1	Female	18	Congenital	Profound	p.Leu184Ser	p.Leu184Ser
CQ‐176‐II‐2	Male	16	Congenital	Profound	p.Leu184Ser	p.Leu184Ser

a NP_076927.1.

### 3.2 Variant identification

Targeted capture and MPS of the three affected individuals yielded an average of 7.1 million reads per sample and a coverage of >92% at 10X. For samples CQ‐176‐II‐1 and CQ‐176‐II‐2, we checked for compound heterozygous or homozygous variants that were shared between siblings. Only a single homozygous variant (c.551T>C; p.Leu184Ser) in *TMPRSS3* was identified. Segregation analysis showed each unaffected parent carried a single copy of the c.511T>C variant (Figure 1a). In GD‐395, we also identified the c.511C>T variant in *TMPRSS3* in a heterozygous state (Figure 2a), with no causative mutations in other known genes associated with HL.

This variant was ultrarare with a MAF of 0.000,22 in East Asians in gnomAD, absent from 300 ethnically matched normal hearing controls and was not known to be disease causing according to the Deafness Variation Database (Azaiez et al., 2018). It was highly conserved and was predicted deleterious by SIFT, Polyphen‐2, and LRT. It had a CADD score of 23.7. Residue Leu184, located in the SRCR domain, was highly conserved across species (Figure 1b). The tertiary structure of the wild‐type protein was compared with the mutant structure predicted by SWISS‐MODEL (Figure 1c). The missense mutation p.Leu184Ser altered the secondary and tertiary structures of the scavenger‐receptor domain.

### 3.3 CNV analysis

We conducted CNV analysis on GD‐395. CNVplex^®^ analysis revealed a heterozygous deletion of exon 11 and part of exon 10 in *TMPRSS3* (Figure 2b), which were confirmed by real‐time PCR using primers designed for exons 7–13 (Figure 2c). In the bam file, manual visualization of exon 10 showed an apparent drop of read‐depth and split‐read mapping (21 of 80 reads) with reads aligning to two different positions in chromosome 21, one part on the forward strand and the other part on the reverse strand (Figure 2d,g). Long‐range PCR (Figure 2e) and Sanger sequencing covering the entire genomic segment revealed a complex genomic rearrangement consisting of a ~ 12 kilo base (kb) inversion (chr21:43800255–43812203) flanked by two deletions and an insertion. On the 5′ end there was an 18 base pair deletion (chr21:43812204–43812221) in intron 2 and a 4 base pair insertion (chr21:43812221‐43812222insCCTG). On the 3′ end there was a ~4kb deletion (chr21:43796202–43800254) spanning part of exon 10 through intron 11 (Figure 2h–i). The detection of the genomic rearrangement in other family members was accomplished by gap‐PCR and agarose gel electrophoresis. The wild‐type allele made an 893 bp product and the mutant allele made a 417bp one. Segregation analysis revealed the genomic rearrangement was in trans with the c.551T>C (Figure 2f).

## 4 DISCUSSION

Here, we performed comprehensive genetic analysis on three cases with prelingual profound ARNSHL. We implicated two variants in *TMPRSS3*, a missense variant and a novel complex genomic rearrangement as the cause of HL in these cases. In the sibling pair from family CQ‐176, we identified a homozygous ultrarare missense variant (c.551T>C) and in GD‐395 we identified the same missense variant in trans with a complex CNV.

The complex genomic rearrangement results in a deletion of exon 11, part of exon 10, and an inversion of exon 3 to exon 9. The two yellow parts get lost accompanied by an insertion of green part when the DNA goes inverted (Figure 2h). The inverted allele possesses an aberrant junction of intron 2 and exon 10. Given the extent of the gene disruption, we expect this rearrangement results in a mutant allele that undergoes nonsense‐mediated decay resulting in null allele. The 5′ end of the inversion falls into mammalian‐wide interspersed repeat 3 of the short interspersed nuclear elements, which may be associated with the CNV mutagenesis during DNA replication.

**Figure 3:**
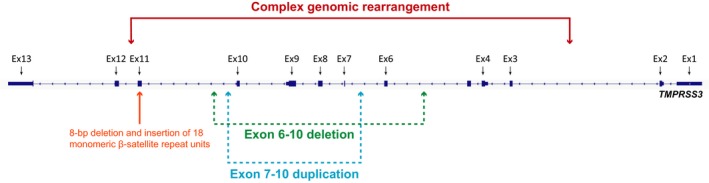
Overview of the copy number variants in *TMPRSS3* reported to date

To date, only four CNV's in *TMPRSS3* have been linked to deafness (Figure 3). An 8‐bp deletion and an insertion of 18 monomeric β‐satellite repeat units in exon 11 were reported in a Palestinian family (Scott et al., 2001). A large deletion of five exons, a homozygous duplication of exon 7–10, and a deletion spanning exons 6–10 all have been reported to cause HL(Shearer et al., 2014; Sloan‐Heggen et al., 2015,2016). To this list, we add a genomic rearrangement consisting of an inversion flanked by two deletions and an insertion.

The missense mutation, p.Leu184Ser, can be defined as a pathogenic variant according to the ACMG guidelines (Richards et al., 2015). It is found in either homozygote or compound heterozygote in trans with the complex genomic rearrangement in different samples. It shows a frequency of 0.000,217,5 in East Asian populations in gnomAD. It is not detected in our 300 controls either. The mutation is predicted to be damaging, deleterious, and conserved by five in silico computational tools, SIFT, Polyphen‐2, LRT, PhyloP, and GERP++, although it is predicted to be a polymorphism in MutationTaster. The CADD PHRED score is 23.7. The SRCR domain contains four cysteine rich motifs and binds to negatively charged molecules such as lipoproteins and sulphate polysaccharides (Fan, Zhu, Li, Ji, & Wang, 2014). The wild‐type Leu184 residue forms a hydrogen bond with Gln144, while the mutant Ser184 residue is predicted to form four hydrogen bonds with Gln144, His186, and Ser187. These additional bonds are expected to impact protein folding resulting in altered intrachain and interchain interactions inside the scavenger‐receptor domain and probably disrupts the binding between the protease and other molecules. Missense variants in this domain have been associated with HL (Table 2 and Table S3). This is the first report linking alterations at residue 184 to deafness.

**Table 2:** Overview of the 77 reported pathogenic variants in *TMPRSS3*

Variants categories	Domain	Variant number	Origin
Missense variants	TM	1	NA
	LDLRA	7	Chinese, Greek, Iranian, Japanese, Pakistani, Polish
	SRCR	10	British, Caucasian, Chinese, Dutch, Indian, Japanese, Pakistani, Polish, Turkish
	Serine protease	29	Chinese, Dutch, German, Indian, Italian, Japanese, Korean, Pakistani, Polish, Tunisian, Turkish
	–	5	Caucasian, German, Iranian, Polish, Turkish
Nonsense variants		8	Chinese, Iranian, Japanese, Pakistani, Palestinian, Turkish
Frameshift variants		6	Chinese, Dutch, Greek, Palestinian, Polish, Slovenian, Spanish, Turkish
Splice site variants		6	Chinese, Dutch, Indian, Korean, Newfoundlander, Pakistani, Polish, Saudi Arabian
Copy number variants		5	Chinese, Iranian, Palestinian

LDLRA: low‐density‐lipoprotein receptor A domain; NA: origin of the variant is not mentioned in the reference; SRCR: scavenger‐receptor cysteine‐rich domain; TM: transmembrane domain.

Variants in *TMPRSS3* are associated with NSHL in more than 20 ethnic groups worldwide. Table 2 summarizes the 77 variants reported to date, classified as missense, nonsense, frameshift, splice site variants, and copy number variants. Based on the locations of the variants, missense variants are further classified into the TM group, the LDLRA group, the SRCR group, the serine protease group, and variants that are not in domains. Detailed information is summarized in Table S3.

In this study, we used a tiered approach to investigate CNVs. The phenotype and family history of GD‐395 was consistent with the ARNSHL. Using MPS, we detected no causative mutations in other HL genes but one heterozygous pathogenic variant in *TMPRSS3*. Therefore, CNV analysis in *TMPRSS3* was considered. We took full advantage of the high‐throughput feature of CNVplex^®^ to screen CNVs in the gene of interest. We then performed real‐time PCR to confirm the CNV. These results highlight the power of using CNVplex^®^ to detect CNV in patients with HL. Detecting this CNV prompted us to reassess MPS data for sequencing reads covering exons 10 and 11. As expected we saw a drop in read‐depth in exon 11. Additionally, we also identified 21 reads that showed split‐mapping in exon 10. Intrigued by this unique mapping event, we sought to resolve the split‐read mapping by direct sequencing. Using long‐range PCR with primers flanking exons 12 and 3 (Figure 2h), we amplified a 16 kb product. Gel electrophoresis showed preferential amplification of a 12 kb product in the proband, whereas the control showed the expected wild‐type 16 kb product (Figure 2e). Breakpoints were identified by Sanger sequencing and confirmed with gap‐PCR (Figure 2f,i). A review of MPS data of 300 controls and >700 affected cases did not identify any other sample with split‐read mapped to exon 10, suggesting this CNV is ultrarare. This study further showcases the importance of comprehensive genetic screening using MPS and the breadth of variants that can be detected using this methodology.

Quantitative analyses, such as multiplex ligation‐dependent probe amplification, CNVplex^®^ analysis and real‐time PCR, are routine methods for detecting CNVs. They can quantify copy numbers and identify abnormality across the genome. However, some structural variations (SVs), such as balanced translocations and inversions, are not involved in abnormal copy numbers and cannot be detected by the quantitative analyses. In addition, quantitative analyses that require high sensitivity in experiments, are both time and labor expensive. As for MPS, the flexibility with design and decreasing cost allow for a cheaper approach to detect CNVs. However, the prerequisite is high levels of sequencing quality and read‐depth. Also, the repeated sequences around the breakpoints, which are very common in CNVs and SVs, severely interfere with reads mapping and identification of the exact mutant sequences. In summary, the quantitative analyses and the MPS are imperfect but critical for CNV detection.

In conclusion, we identified a novel complex genomic rearrangement and a novel missense mutation in *TMPRSS3* that cause HL. This work highlights the need for comprehensive genetic testing that includes CNV detection for HL.

## CONFLICT OF INTEREST

None declared.
