# Supplementary material for: Identification of a complex genomic rearrangement in TMPRSS3 by massively parallel sequencing in Chinese cases with prelingual hearing loss
Source: Mol Genet Genomic Med. 2019 Apr 23;7(6):e685. doi: 10.1002/mgg3.685 (PMC6565588; doi:10.1002/mgg3.685)
Supplement: Supplementary file 1 [file MGG3-7-e685-s001.docx]

## Supporting Information

**Table S1. 50 probes used in CNVplex® analysis**

| Locus Name | Probe binding sequences | Length | Chromosome position |
| --- | --- | --- | --- |
| TMPRSS3-10kb_1 | AAAACCACGATGGCAGCACTCAGGGGACAGGCGCTAGAACTTC | 43 | chr21:43826237-43826279 |
| TMPRSS3-2kb_1 | GCGTCCAGGCAGTCCTTGATTAAGACAGTCTTACTTTCTCCGTCCCA | 47 | chr21:43818253-43818299 |
| TMPRSS3-exon1_1 | AATGTAGATGGCACCACGGAAGAGATAGTAGGCCACAGTGTTACTGGCTTC | 51 | chr21:43816070-43816120 |
| TMPRSS3-exon1_2 | CATAAACACAGCCCTTTCCTGGCTCACACGGGCATGACCTAATTAAG | 47 | chr21:43816122-43816168 |
| TMPRSS3-exon2_1 | CCACAGGGACAGTCAGTCACATTGGTCACCTACATGAGGGTATGGGCA | 48 | chr21:43815352-43815399 |
| TMPRSS3-exon2_2 | AAATCACAGAGTCCTCACCTGGGTCCACTTACCTGGTGCAACAGGA | 46 | chr21:43815401-43815446 |
| TMPRSS3-exon3_1 | AAAACTTCAATGGCAGCAGTGACAGGATCTGTGCAGCAACAGCATC | 46 | chr21:43810096-43810141 |
| TMPRSS3-exon3_2 | AAAAGGAAGAGCAGAAAGCCACAGAACCTGACTTGGAGTGCCATGA | 46 | chr21:43810159-43810204 |
| TMPRSS3-exon4_1 | AGCTCGATACACTTAAAGGATGAGCGACATCTGTACTTCCCTGAGCAGTCG | 51 | chr21:43809097-43809147 |
| TMPRSS3-exon5_1 | TGGGCACAGGCAACATTTGCGTAGTGACCCTTCCAGTCATCGG | 43 | chr21:43808527-43808569 |
| TMPRSS3-exon5_2 | GCACATGGTCTTCCACGAAGCAGCTGTGAACACCTGGAGCACG | 43 | chr21:43808571-43808613 |
| TMPRSS3-exon6_1 | ACTGAGTGGTGTAATGCAGTCACCTTGTCATCTGGCAAGAGGTGATCG | 48 | chr21:43805527-43805574 |
| TMPRSS3-exon6_2 | GAAAGACAGACCCGACGTGGTAACTTTACACTTTGAAGCATTCAACCGATG | 51 | chr21:43805658-43805708 |
| TMPRSS3-exon7_1 | GAGAGGACTCTGAGGGCAAGGAGATAGGACTTAGCATGTGCTTGCTCC | 48 | chr21:43804000-43804047 |
| TMPRSS3-exon7_2 | GCACTGCAAGGTAACCACGTGGCCAGAGGCACATCCCTCCCTAA | 44 | chr21:43804083-43804126 |
| TMPRSS3-exon8_1 | CCACTGCGAGAGCAAGGACATGTTTCCACCCACGATGCGT | 40 | chr21:43803240-43803279 |
| TMPRSS3-exon8_2 | TATGGAGGGGAACAAAGGCTTGTGGGTCCACCCTGCAGACTT | 42 | chr21:43803309-43803350 |
| TMPRSS3-exon9_1 | CCATTGAACGTGAGTGGCCCGGCCAGCTTCATAAGGGCGA | 40 | chr21:43802173-43802212 |
| TMPRSS3-exon9_2 | CAAGTGGGATGGGGCTGGATTGTCCAACAGGGAAACTAGACCCA | 44 | chr21:43802268-43802311 |
| TMPRSS3-exon10_1 | CCATCCTGACGTCCAGCACACTTTTCCATCGGGGAAGTTCTCTT | 44 | chr21:43800245-43800288 |
| TMPRSS3-exon10_2 | CAATTGAAGCAGGAGTCCGAGAAAACAATCTCATCTATGCTTCTGCCTCTG | 51 | chr21:43800352-43800402 |
| TMPRSS3-exon11_1 | GGGGAGATGATGCCACCGTACACGTCCCTGTGGTTGCAGA | 40 | chr21:43796699-43796738 |
| TMPRSS3-exon11_2 | GGAGGCGTCACCTGCTTCAAAGTGAGTGAGGGGATGTGTGTGA | 43 | chr21:43796782-43796824 |
| TMPRSS3-exon12_2 | GCTCCCACTAACTTCCACAGCCTCCTCTCTTGACACACCAGGGG | 44 | chr21:43795919-43795962 |
| TMPRSS3-exon13_1 | GTGACTGCAGTTCTGCACTTCTGGGCTGGTGCGTCTTTTCTGAGTG | 46 | chr21:43792185-43792230 |
| TMPRSS3-exon13_2 | TGCTACTGGTGCCGGAACTCAGAGCTCCAAGGGTGTCTGCTCGT | 44 | chr21:43792740-43792783 |
| 2p-1_1 | GGCTTTCTTCCCATCCCAGTCAGCCTGAAAAATTGTCTCTATCAGGGAAA | 50 | chr2:84500198-84500247 |
| 3p-2_2 | GCCACTGGGGCTTTAGGAATGGTACTTCGCAGCTCAGTGCAGCC | 44 | chr3:87027376-87027419 |
| 3p-3_2 | GCAAGTAGGATTGCAAAGGAGGAGGTGAATGGCCAAGTCCTTTTGGAG | 48 | chr3:88040548-88040595 |
| 3q-3_1 | GATGAAGGGTCTTTGGCTTCCTTTAGACCAAGAAGGGTAGTGAGATTCTAGCAGCC | 56 | chr3:99043643-99043698 |
| 4q-3_1 | TTCATCTGGATCCATGACGATGGACAAGTAGCCTGTCTTCAGTTCCCCTC | 50 | chr4:55964383-55964432 |
| 5p-2_1 | CCAAGAGGGAAGGCAGGCAGAAGACAAATAGCAGCGGTGGTGCC | 44 | chr5:44387750-44387793 |
| 6p-3_1 | TGCCTAGCTGTGTGACATGATTGGCATCCCCAGTGTGGGACCATG | 45 | chr6:51589994-51590038 |
| 7p-2_1 | GCTGGGGCCTTTTTTCAGCATCCTGGAGCCCTGAACAAGTACAAATG | 47 | chr7:48085197-48085243 |
| 8p-1_2 | CCCAATGGTTGCAAGAGGTTCCCTAGGACTAGGGTCTTGCATAGAGCCTTC | 51 | chr8:37478885-37478935 |
| 8p-2_2 | CACTGTGATTCAGCAGTGAAGTCCTGCTCTCTCGAGCTAACATTGTGGAGGG | 52 | chr8:38646986-38647037 |
| 8q-3_1 | TTTTCCCTGTTACATAGTGCTGGGCAGCCTCTGGGGGCCTCACA | 44 | chr8:48436239-48436282 |
| 9p-2_2 | AGATTATTGAAGGTGGCCCAGGCTCAGGGTGGGGGAAGTGGC | 42 | chr9:36714558-36714599 |
| 9q-3_2 | GTGCCGGAGAAGATGATTCATGACTCTAGCCCTGCTTTTTCTCTCCCG | 48 | chr9:75301852-75301899 |
| 10p-1_1 | CAGGCATAACAAACAGCTCAATTCCCACGCATGCAGCCCTCTCCA | 45 | chr10:31119656-31119700 |
| 11p-1_1 | GCAGCAAAGGCACATTTACTCCGTATTGGAGTTCATCATGCCCAGGG | 47 | chr11:41893549-41893595 |
| 12p-3_2 | CTGGAGTGAGGGGAAGAAGCTGTTACAGAAGTGGAATGGTTTCTGGTGG | 49 | chr12:27952126-27952174 |
| 12q-1_1 | CAGCGCTCATTCTTTAGTCTCCGAACAGAATGACAGGGTTTGTGAAGTCG | 50 | chr12:41221686-41221735 |
| 14q-2_2 | TGGACAGCTTGTCCCACCTCCCTCATGGAGCCCAAGAAGGATTCA | 45 | chr14:23070671-23070715 |
| 16p-3_1 | CCACTGTCCCCTTCATCTGATGATTTCCCCAAAGATGCTGCCTGTAGATG | 50 | chr16:27768781-27768830 |
| 16q-3_2 | CCACCCCAGGAATATCCAATTAGCAAATCATGATCCGGCCCTCCA | 45 | chr16:51483383-51483427 |
| 18p-1_1 | CGTACAGACTTTAGGGAGCCTGTGTTCAGTGTGGCCTATAAGGATTTGGGTATGG | 55 | chr18:13437524-13437578 |
| 19q-1_2 | GCTTCCTGGGAGCAGAATTGTTCTTTTTCTTCCCATAGTGTGCCACTGC | 49 | chr19:30737413-30737461 |
| 20p-1_1 | AAGGCCAGGCCTGTATTTAGTTAGTGGTAATGATCTCGTTAGGGGTGTGAATGG | 54 | chr20:20868408-20868461 |
| 20q-1_2 | GCCACCCTCCAGTAGCCTTTTCCTGGTGGTTCTGCATTGTACCTGAGAAA | 50 | chr20:35866127-35866176 |

**Table S2. Primers designed for real-time PCR and subsequent experiments**

| Primer name | Forward | Reverse | Product position |
| --- | --- | --- | --- |
| TMPRSS3_In2 | TCTCCTCGCATTCCATGTCT | CCACTAGCACTCTTCCCTCC | chr21:43812052-43812441 |
| TMPRSS3_Ex7 | CCGCTTTAGGGAGGGATGTG | TGAGGGCAAGGAGATAGGAC | chr21:43804010-43804131 |
| TMPRSS3_Ex8 | AAACATGTCCTTGCTCTCGC | ATGACCCAGGAGTGAACAGG | chr21:43803093-43803264 |
| TMPRSS3_Ex9 | AATGCCAAGACGACCTCAGA | TCCCATCACCCTGCTTGTTT | chr21:43802461-43802585 |
| TMPRSS3_Ex10 | AGATTGTTTTCTCGGACTCCTG | TCCCGAGCAGCTGACATG | chr21:43800083-43800382 |
| TMPRSS3_In10 | TAGTTTCCAACCCACCCACA | AGGAAGGGGAGCGTAGATTG | chr21:43798150-43798285 |
| TMPRSS3_Ex11 | CCTTCTCACACACATCCCCT | GGAGATGATGCCACCGTACA | chr21:43796701-43796829 |
| TMPRSS3_Ex12 | AGAAAGCAATCTCGCATGGC | AGCCTCCTCTCTTGACACAC | chr21:43795937-43796038 |
| TMPRSS3_Ex13 | ATGAAGACAGCCCGATCCTC | GCTTGAAGGTTGTGCTGGAA | chr21:43792692-43792831 |
| RPP30 | GGGTCAGTAGTAGGGAGTGAG | TCGGTCAATCGCCTTCACAG | chr10:92654428-92654543 |
| COBL | GCAAGGCACAGAAGTGTTTGAG | GCTCCTCCACTGCCTACAACG | chr7:51357169-51357330 |

**Table S3. Overview of the 77 variants in *TMPRSS3* reported to date, including those identified in this study**

| **Variants categories** | **Domain** | **DNA change**  **(NM_024022.2)** | **Amino acid change**  **(NP_076927.1)** | **Origin** | **Published reference (PMID)** |
| --- | --- | --- | --- | --- | --- |
| Missense variants | - | c.1343T>C | p.Met448Thr | Polish | 28566687 |
|  | Serine protease | c.1306C>G | p.Arg436Gly | Polish | 28566687 |
|  |  | c.1306C>T | p.Arg436Cys | NA | 26969326 |
|  |  | c.1291C>T | p.Pro431Ser | Italian | 24657061 |
|  |  | c.1286A>G | p.Asn429Ser | NA | 26969326 |
|  |  | c.1276G>A | p.Ala426Thr | Dutch, Polish | 12920079,21786053,28566687 |
|  |  | c.1273T>C | p.Cys425Arg | Pakistani | 21534946 |
|  |  | c.1250G>A | p.Gly417Glu | Chinese | 28695016 |
|  |  | c.1244T>C | p.Leu415Ser | Chinese | 28695016 |
|  |  | c.1219T>C | p.Cys407Arg | Pakistani | 11424922,12920079,15447792 |
|  |  | c.1211C>T | p.Pro404Leu | Tunisian, Turkish | 11462234,12920079,16021470 |
|  |  | c.1204G>A | p.Gly402Arg | Chinese | 28695016 |
|  |  | c.1159G>A | p.Ala387Thr | Japanese | 24130743,25770132 |
|  |  | c.1156T>C | p.Cys386Arg | Indian | 24416283 |
|  |  | c.1151T>G | p.Met384Arg | Chinese | 28695016 |
|  |  | c.1129G>A | p.Gly377Ser | Turkish | 26226137 |
|  |  | c.1028G>T | p.Trp343Leu | Japanese | 25770132 |
|  |  | c.1025G>A | p.Gly342Glu | Turkish | 21117948 |
|  |  | c.1019C>G | p.Thr340Arg | Italian | 24657061 |
|  |  | c.974T>A | p.Leu325Gln | Polish | 28566687 |
|  |  | c.916G>A | p.Ala306Thr | German, Dutch, Korean, Chinese | 17551081,21786053,24526180,28246597,28695016 |
|  |  | c.913A>T | p.Ile305Phe | Turkish | 26226137 |
|  |  | c.809T>A | p.Ile270Asn | Chinese | 28695016 |
|  |  | c.771C>G | p.His257Gln | Japanese | 25770132 |
|  |  | c.767C>T | p.Ala256Val | Pakistani | 21534946 |
|  |  | c.763G>T | p.Ala255Ser | Chinese | 27610647 |
|  |  | c.753G>C | p.Trp251Cys | Tunisian | 11462234,12920079 |
|  |  | c.743C>T | p.Thr248Met | Korean | 24526180 |
|  |  | c.727G>A | p.Gly243Arg | Indian | 24416283 |
|  |  | c.726C>G | p.Cys242Trp | Pakistani | 24949729 |
|  | - | c.647G>A | p.Arg216His | Iranian | 26445815 |
|  | - | c.647G>T | p.Arg216Leu | Turkish | 16021470 |
|  | - | c.646C>T | p.Arg216Cys | German, Caucasian | 17551081/22975204 |
|  | - | c.616G>T | p.Ala206Ser | Turkish | 26226137 |
|  | SRCR | c.595G>A | p.Val199Met | Dutch | 21786053 |
|  |  | c.581G>T | p.Cys194Phe | Pakistani | 11424922,12920079,15447792 |
|  |  | c.554A>T | p.His185Leu | Japanese | 23967202 |
|  |  | c.551T>C | p.Leu184Ser | Chinese | **This study** |
|  |  | c.535G>A | p.Asp179Asn | Tibetan | 25474651 |
|  |  | c.436G>A | p.Gly146Ser | Turkish | 26226137 |
|  |  | c.413C>A | p.Ala138Glu | British, Dutch, Caucasian, Polish | 16283880,21786053,22975204,28566687 |
|  |  | c.399G>C | p.Trp133Cys | Turkish | 26226137 |
|  |  | c.371C>T | p.Ser124Leu | Polish | 28566687 |
|  |  | c.346G>A | p.Val116Met | Indian | 24416283 |
|  | LDLRA | c.326G>A | p.Arg109Gln | Chinese | 24853665 |
|  |  | c.325C>T | p.Arg109Trp | Pakistani, Iranian, Polish | 11424922,12920079,26445815,28566687 |
|  |  | c.316C>T | p.Arg106Cys | Japanese, Chinese | 23967202,28246597 |
|  |  | c.310G>A | p.Glu104Lys | Pakistani | 21534946 |
|  |  | c.308A>G | p.Asp103Gly | Greek | 11907649,12920079 |
|  |  | c.280G>A | p.Gly94Arg | Japanese | 23967202,25770132 |
|  |  | c.218G>A | p.Cys73Tyr | Polish | 28566687 |
|  | TM | c.188T>G | p.Leu63Arg | NA | 26969326 |
| Nonsense variants | | c.1192C>T | p.Gln398Ter | Turkish | 16021470 |
|  |  | c.677C>A | p.Ser226Ter | Chinese | 24853665 |
|  |  | c.607C>T | p.Gln203Ter | Japanese | 24130743,25770132 |
|  |  | c.582T>A | p.Cys194Ter | Palestinian | 19888295 |
|  |  | c.310G>T | p.Glu104Ter | Pakistani | 21534946 |
|  |  | c.271C>T | p.Arg91Ter | Turkish | 26226137 |
|  |  | c.115C>T | p.Gln39Ter | NA | 26969326 |
|  |  | c.46C>T | p.Arg16Ter | Iranian | 27344577 |
| Frameshift variants | | c.999delC | p.Asp334MetfsTer24 | Polish | 28566687 |
|  |  | c.989delA | p.Glu330GlyfsTer28 | Palestinian | 16460646 |
|  |  | c.579dupA | p.Cys194MetfsTer17 | Slovenian, Polish | 26036852,28566687 |
|  |  | c.208delC | p.His70ThrfsTer19 | Spanish, Greek, Dutch, Slovenian, Polish | 11907649,21786053,26036852,28566687 |
|  |  | c.36dupC | p.Phe13LeufsTer10 | Turkish | 23226338,26226137 |
|  |  | c.36delC | p.Phe13SerfsTer12 | Chinese | 28246597 |
| Splice site variants | | c.1195-1G>C |  | Saudi Arabian | 21726435 |
|  |  | c.953-5A>G |  | Polish | 28566687 |
|  |  | c.783-1G>A |  | Korean | 29072634 |
|  |  | c.782+8insT |  | Newfoundlander | 15447792 |
|  |  | c.782+2T>A |  | Polish | 28566687 |
|  |  | c.323-6G>A |  | Pakistani, Dutch, Indian, Chinese | 11137999,21786053,24416283,28695016 |
| Copy number variants | | 8-bp deletion and insertion of 18 monomeric β-satellite repeat units |  | Palestinian | 11137999 |
|  |  | 5 exons deletion |  | NA | 24963352 |
|  |  | Exon 7-10 duplication |  | Iranian | 26445815 |
|  |  | Exon 6-10 deletion |  | NA | 26969326 |
|  |  | complex genomic rearrangement |  | Chinese | **This study** |
